# Focus on Glucagon-like Peptide-1 Target: Drugs Approved or Designed to Treat Obesity

**DOI:** 10.3390/ijms26041651

**Published:** 2025-02-14

**Authors:** Jiahua Zhang, Jintao Wei, Weiwen Lai, Jiawei Sun, Yan Bai, Hua Cao, Jiao Guo, Zhengquan Su

**Affiliations:** 1 Guangdong Engineering Research Center of Natural Products and New Drugs, Guangdong Provincial University Engineering Technology Research Center of Natural Products and Drugs, Guangdong Pharmaceutical University, Guangzhou 510006, China; 13188236390@163.com (J.Z.); startwjt@163.com (J.W.); laiweiwengy@163.com (W.L.); sunjw0724@163.com (J.S.); 2 Guangdong Metabolic Disease Research Center of Integrated Chinese and Western Medicine, Key Laboratory of Glucolipid Metabolic Disorder, Ministry of Education of China, Guangdong TCM Key Laboratory for Metabolic Diseases, Guangdong Pharmaceutical University, Guangzhou 510006, China; 3 School of Public Health, Guangdong Pharmaceutical University, Guangzhou 510310, China; angell_bai@163.com; 4 School of Chemistry and Chemical Engineering, Guangdong Pharmaceutical University, Zhongshan 528458, China; caohua@gdpu.edu.cn

**Keywords:** obesity, double and triple glucagon-like peptide-1 (GLP-1) targeted drugs, hypothalamus, gastric inhibitory peptide, fecal bacteria transplantation (FMT)

## Abstract

Obesity is closely related to metabolic diseases, which brings a heavy burden to the health care system. It is urgent to formulate and implement effective treatment strategies. Glucagon-like peptide-1 (GLP-1) is a protein with seven transmembrane domains connected by type B and G proteins, which is widely distributed and expressed in many organs and tissues. GLP-1 analogues can reduce weight, lower blood pressure, and improve blood lipids. Obesity, diabetes, cardiovascular diseases, and other diseases have caused scientists’ research and development boom. Among them, GLP-1R agonist drugs have developed rapidly in weight-loss drugs. In this paper, based on the target of GLP-1, the mechanism of action of GLP-1 in obesity treatment was deeply studied, and the drugs approved and designed for obesity treatment based on GLP-1 target were elaborated in detail. Innovatively put forward and summarized the double and triple GLP-1 targeted drugs in the treatment of obesity with better effects and less toxic and side effects, and this can make full use of multi-target methods to treat other diseases in the future. Finally, it is pointed out that intestinal flora and microorganisms have many benefits in the treatment of obesity, and fecal bacteria transplantation may be a potential treatment for obesity with less harm to the body. This article provides some promising methods to treat obesity, which have strong practical value.

## 1. Introduction

Glucagon-like peptide-1 (GLP-1) is a type B, G-protein-linked seven transmembrane domain protein that is widely distributed in many organs or tissues, including the central nervous system, gastrointestinal tract, cardiovascular system, liver, and fat tissue. It can be expressed in the pancreas, lungs, heart, kidneys, hypothalamus, and stomach [1].

When GLP-1 analogues are used in the treatment of type 2 diabetes, the hypoglycemic effect is significant, and the effects of weight loss, blood pressure reduction, and lipid profile improvement are also achieved [2]. The market size of GLP-1R agonists (GLP-1RA) continues to grow. It has triggered a frenzy of research and development of small molecule GLP-1R agonists for various conditions, such as obesity, diabetes, Metabolic dysfunction-associated steatotic liver disease (MASLD)/Nonalcoholic fatty liver disease (NAFLD), Metabolic dysfunction-associated steatohepatitis (MASH)/Nonalcoholic steatohepatitis (NASH) [3], metabolic disorders, Alzheimer’s disease, Parkinson’s disease, cardiovascular disease and other drugs [4,5]. Among them, GLP-1R agonist drugs have developed rapidly in weight reduction drugs.

**Figure 1:**
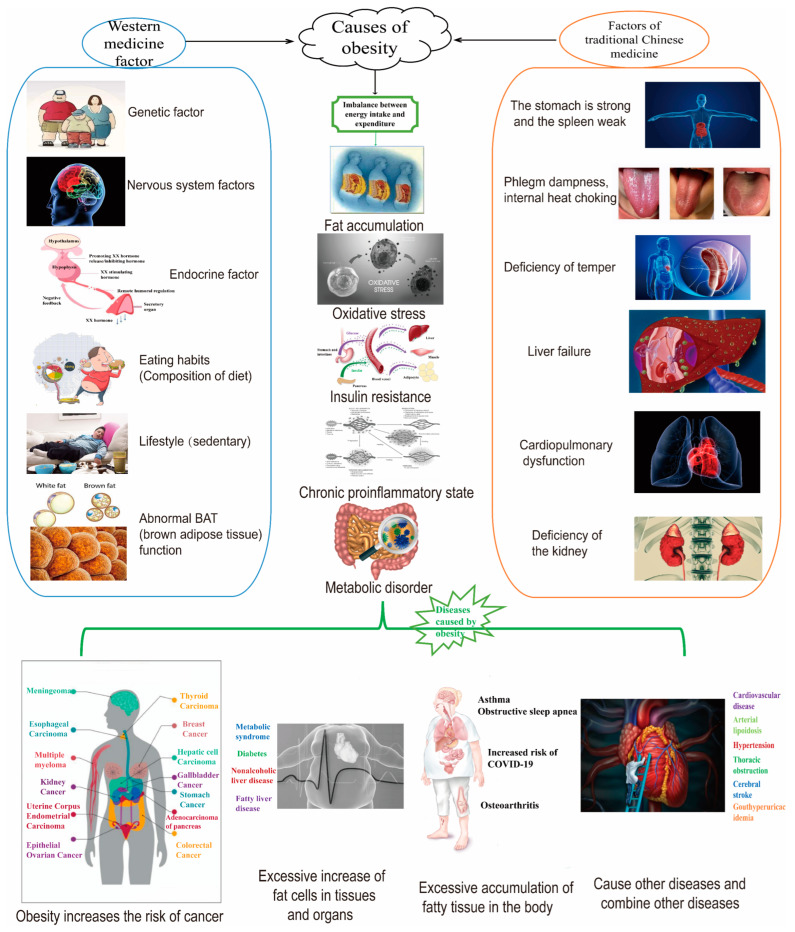
Obesity causes various diseases (Traditional Chinese medicine and Western Medicine perspectives).

With approximately 40% of adults worldwide overweight or obese, the large number of patients and the characteristics of long-term medication for chronic diseases make obesity drugs the second largest market in the world. Obesity is a relatively complex chronic metabolic disease caused by the interaction of multiple factors [6,7,8]. Currently, it is believed that the main risk factors of obesity are genetic factors, endocrine hormone imbalance, excessive diet, and sedentary lifestyle [9,10,11,12]. From the perspective of traditional Chinese medicine, the pathogenesis of obesity is that the stomach is strong while the spleen is weak, which produces phlegm and dampness, leading to qi stagnation, blood stasis, and internal heat blockage. The disease location is mainly in the spleen, stomach, and muscles, closely related to kidney deficiency, heart and lung dysfunction, and liver dysfunction (As shown in Figure 1). At present, the vast majority of obesity is due to excessive diet and sedentary lifestyle [13], which leads to the imbalance of energy intake and consumption. The human body has been in a state of chronic pro-inflammatory and metabolic disorders for a long time [14]. Studies have shown that obesity also increases the risk of developing Corona Virus Disease 2019 (COVID-19) [15].

There are two main steps to treat obesity: reducing calorie intake and increasing calorie expenditure. GLP-1, as one of the important targets in the treatment of obesity, has some characteristics, such as a remarkable curative effect, short half-life, and easy clearance [16]. The development of GLP-1 long-acting receptor agonists is a research hotspot at home and abroad. Recently, the well-known academic journal Science announced that glucagon-like peptide-1 agonist was selected as Science’s 2023 Breakthrough of the Year [17]. This marks an important milestone in the long fight against obesity and its related complications.

## 2. Mechanism of Action of GLP-1 Target in the Treatment of Obesity

**Figure 2:**
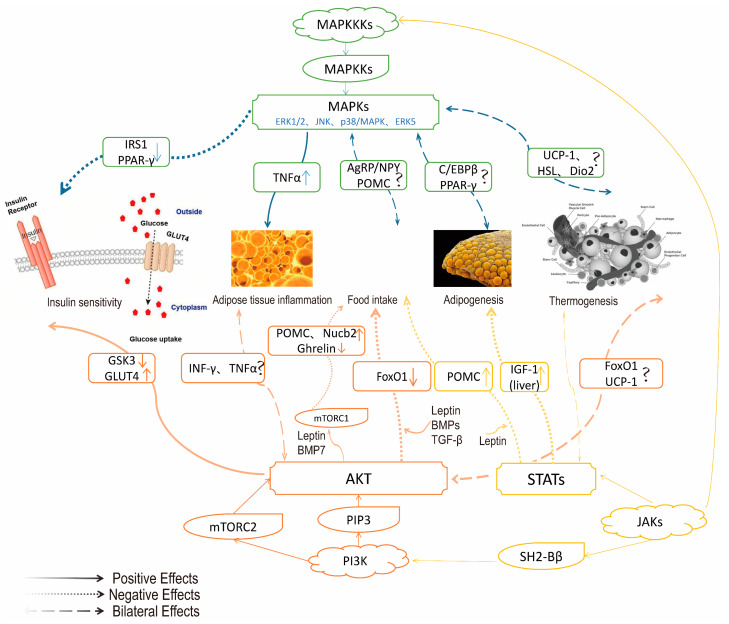
Application of MAPK, PI3K, and JAK/STAT signaling pathways in the pathogenesis of obesity. Photo Note: Mitogen-activated protein kinase (MAPK) is a key mediator of signal transduction in mammalian cells. The MAPK signaling pathway consists of a three-layer kinase cascade consisting of MAPK kinase kinases (MAPKKK), MAPK kinases (MAPKKs), and MAPK. ERK, JNK, p38 MAPK, ERK5, and other MAPKs play a complex role in appetite regulation, lipogenesis, and glucose homeostasis [25]. JNK and p38 have similar functions and are related to inflammation, apoptosis, and growth. ERK mainly involves tube cell growth and differentiation, and its upstream signal is the well-known Ras/Raf protein, ERK5, and ERK1/2 pathways, which are similar. MAPK signaling pathway plays different roles in adipose tissue browning and thermogenesis. Note: ↑ indicates an increase in content, and ↓ indicates a decrease in content. “?” Indicates that the research is uncertain.

**Figure 3:**
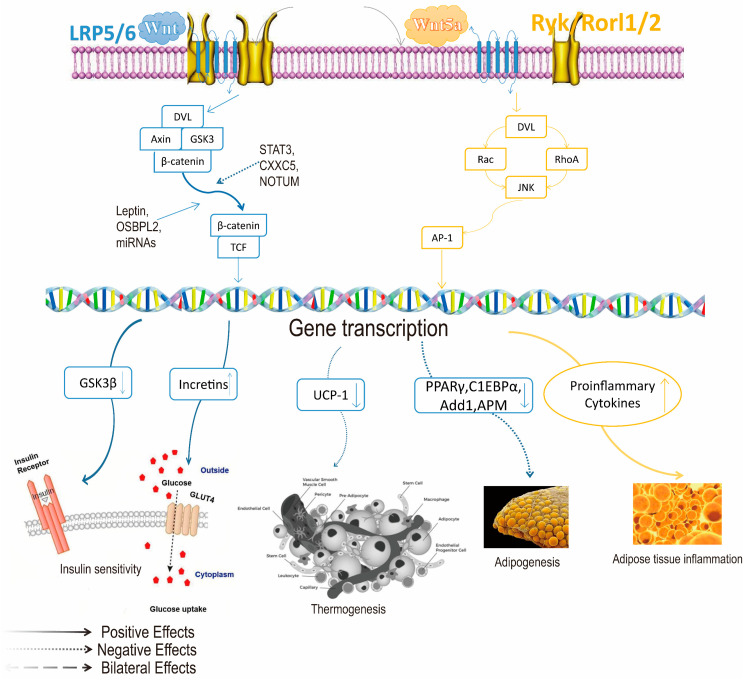
The Wnt/β-catenin pathway in the pathogenesis of obesity. Photo Note: The Wnt/β-catenin pathway is a normative pathway in Wnt signaling, and the activation/inhibition of the Wnt signaling pathway leads to different roles in the pathogenesis of obesity [26], which is determined by specific action pathways. The Wnt/β-catenin pathway is thought to inhibit adipogenesis and the development of obesity. Wnt/β-catenin can inhibit the expression of adipocyte-related genes (including PPARγ and fatty acid synthase) and inhibit adipogenesis [27]. But Wnt/β-catenin signaling plays a complex role in different fat pools, different diets, and different stages of fat production. The Wnt/β-catenin pathway also affects insulin action and systemic glucose homeostasis. Promoting this signaling helps regulate energy homeostasis. After activation of the Wnt protein, beta-catenin is released and enters the nucleus as a transcriptional co-activator of T-cell factor (TCF) to regulate the transcription of target genes. Note: ↑ indicates an increase in content, and ↓ indicates a decrease in content.

GLP-1 is mediated by peripheral or central signaling and acts by activating the GLP-1 receptor (GLP-1Rs). Peripheral GLP-1 may reduce insulin resistance and weight loss by enhancing PKA (protein kinase A) activity, reducing endoplasmic reticulum stress, and improving β cell function [18]. Improve insulin sensitivity in peripheral tissues by inhibiting AMPK-related pathways. By interacting with pathways such as PI3K, MAPK [19], and Wnt/β-catenin, upregulation of PPARγ and FABP4 promotes preadipocyte differentiation and inhibits adipogenesis in mature adipocytes (as shown in Figure 2 and Figure 3). GLP-1 signaling is a regulator of fat formation [20]. Adipocyte differentiation can counteract the negative metabolic effects of obesity. GLP-1R activation can directly promote lipolysis and fatty acid oxidation by upregulating Sirt1 expression in differentiated 1T3-L3 adipocytes [21] and participate in thermogenic regulation by inhibiting BMP4-related signaling pathways, inducing the expression of thermogenic genes such as UCP1. GLP-1 from the gut can also transmit information upward to the central nervous system via the vagus nerve, which in turn inhibits vagus activity and stomach emptying, increasing satiety [22]. GLP-1 is a type of intestinal insulin and is regulated by classical satiety factors [23].GLP-1R in the brain is not essential for the physiological control of glucose regulation, but the central role of GLP-1R signaling cannot be ignored; it plays a key role in weight loss and may be a prime target for obesity and other metabolic diseases. Activation of GLP-1R has a powerful effect on the regulation of appetite, gastric motivity [24], glucose, lipid metabolism, and even body heat production.

Natural GLP-1 is easily degraded by dipeptidyl peptidase and loses its activity in vivo. In order to make GLP-1 better applied in clinics, drug developers have modified its structure and developed a series of GLP-1 receptor agonists. GLP-1R agonists involve the central nervous system, peripheral target organs, and peripheral tissues. They act mainly through the central nervous system to control hunger and appetite and through the hypothalamus to affect the rate of energy consumption, regulate the secretion of hormones related to energy storage, and then suppress appetite. They can also act on the gastrointestinal tract to delay gastric emptying, reduce food intake, and then reduce weight. By combining with GLP-1 to stimulate insulin secretion, they inhibit glucagon secretion, promote glucose metabolism, reduce blood sugar, improve blood lipid profile, regulate blood lipids, protect cardiovascular function, and reduce the risk of cardiovascular diseases. In addition, the risk of hypoglycemia is low while reducing weight and blood sugar [4].

**Figure 4:**
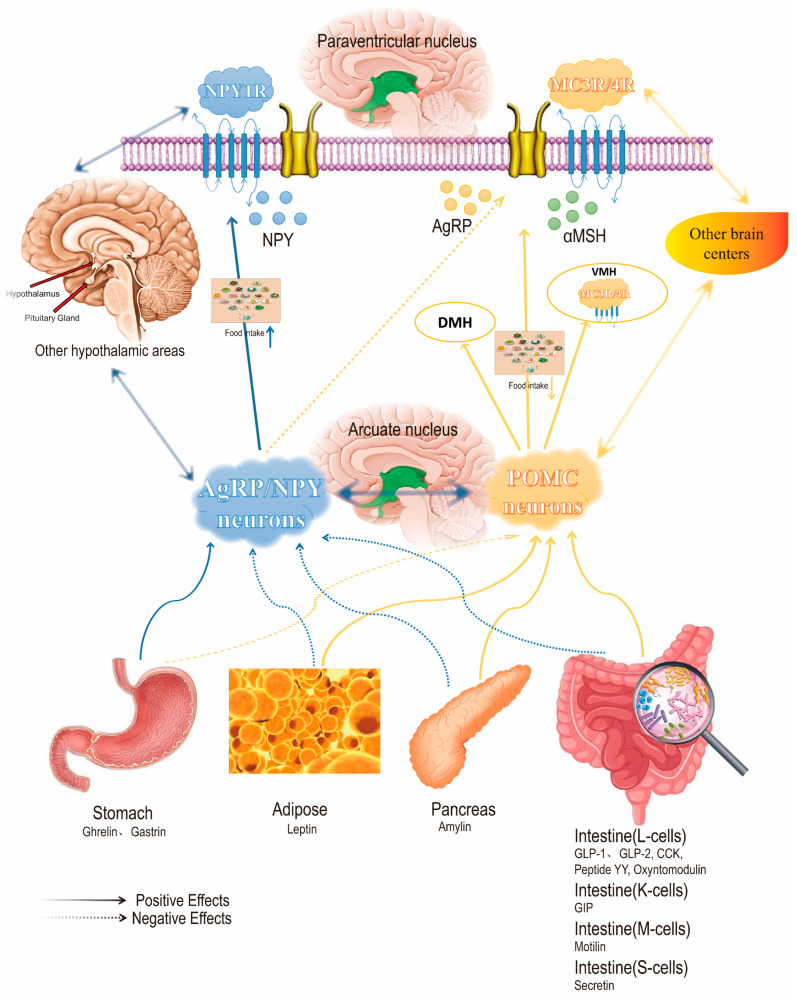
GLP-1 targets regulate appetite through the hypothalamus, thereby treating obesity [29]. Note: ↑ indicates an increase in content, and ↓ indicates a decrease in content.

GLP-1R agonists play a key role in the hypothalamus, the connection between the brain stem and various regions of the forebrain. In the signal integration of energy flux, the most critical hypothalamic nucleus is the arcuate nucleus (also known as the arcuate nucleus, ARC), which is regulated by various neurotransmitters/hormones, receives signal input from multiple parts of the central nervous system, integrates and sends information about appetite. Proopiomelanocortin (POMC) neurons and AgRP/NPY neurons are key neurons in the hypothalamus that regulate appetite and play opposite roles in appetite control. POMC neurons promote satiety, while AgRP neurons promote hunger [28]. Genetic mutations or obesity can cause damage to POMC neurons, disrupting the energy balance and increasing food intake. But we cannot simply think that activating the POMC neurons and destroying the AgRP neurons can achieve the effect of weight loss, as anorexia and lack of energy can be life-threatening; POMC and AgRP neurons together constitute the homeostasis of appetite regulation. It is not the presence of AgRP neurons that causes obesity but an imbalance in the system. The system relies on continuous signal integration and bidirectional crosstalk between the brain and the peripheral primary feeding center. Reasonable adjustment of the balance of this system, in the case of no damage to health, to achieve the purpose of efficient weight loss. Through understanding the neurons and hormones related to appetite, this paper understands that appetite is a normal steady-state regulation and an instinct for survival (As shown in Figure 4).

**Figure 5:**
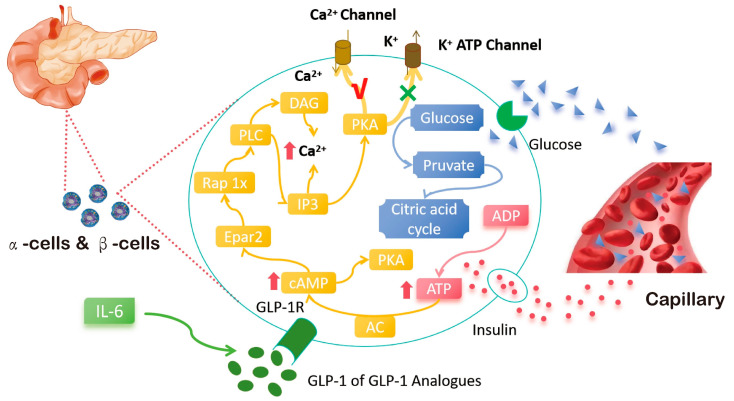
GLP-1 targets and regulates insulin, controlling blood glucose. Notes: GLP-1 can actvate adenylate cyclase (AC) after binding with GLP-1R, and the activated AC will stimulate the conversion of ATP to cyclic adenosine phosphate (cAMP) and increase the concentration of cAMP. cAMP further activates protein kinase A (PKA) and guanine nucleotide exchange factor (Epac2). On the one hand, the activated PKA can close the ATP-dependent K^+^ channel and depolarize the cell membrane; on the other hand, it can activate the voltage-dependent Ca^2+^ channel to make Ca^2+^ flow in and generate an action potential. PKA can also promote Ca^2+^ release by activating inositol triphosphate (IP3). Activated Epac2 can further activate Ras protein 1(Rap1) and phospholipase C (PLC), thereby activating the IP3 and diacylglycerol (DAG) pathways and promoting Ca^2+^ release. All of these pathways lead to an increase in intracellular Ca^2+^ concentration, which promotes mitochondrial synthesis of ATP, allowing insulin particles to be released into the bloodstream.

GLP-1R agonists have a hypoglycemic effect second only to insulin and have the advantages of low risk of hypoglycemia, significant weight loss effect, and cardiovascular benefits. IL-6 is a cytokine that has multiple effects on metabolism and can improve β cell function and glucose homeostasis by affecting GLP-1 upregulation [30]. In islet β cells, GLP-1 depolarizes the cell membrane by binding to GLP-1R, resulting in increased intracellular Ca^2+^ levels and intracellular insulin release, as shown in Figure 5. GLP-1 receptor agonists stimulate insulin secretion and reduce glucagon secretion in a glucose-dependent manner, regulating blood sugar and reducing the risk of hypoglycemia [31].

## 3. Overview of GLP-1 Targeted Drugs Approved or Designed to Treat Obesity

**Figure 6:**
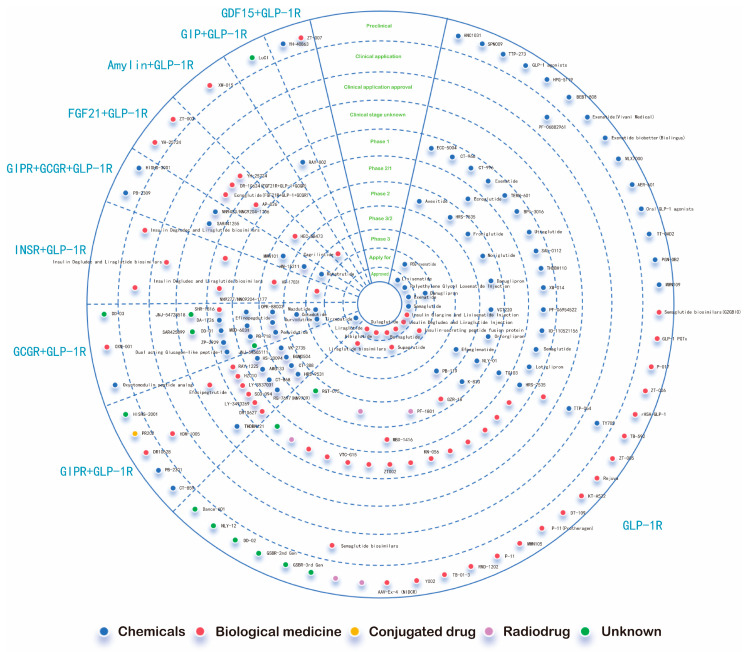
Most GLP-1 targeted drugs.

At present, there are 13 kinds of GLP-1R agonists in the global market. There are seven species in the application stage, 21 species in the phase III clinical trial, 26 species in the phase II clinical trial, 38 species in the phase I clinical trial, 99 species in the preclinical trial, 505 species in the drug discovery stage, and 106 species have no follow-up progress report (Figure 6 indicates the names of some drugs.). Among them, the GLP-1R agonists approved for sale in the world are: Exenatide (Byetta), Exenatide LAR (Bydureon), Semaglutide (Ozempic/Rybelsus), Tirzepatide (Mounjaro), Liraglutide (Victoza), Albiglutide (Tanzeum), Dulaglutide (Tanzeum), Trulicity, Lixisenatide (Adlyxin), Beinaglutide (HYBR-014), Polyethylene Glycol Loxenatide Injection (PEX-168), Insulin Degludec/Liraglutide (IDegLira), Insulin Glargine and Lixisenatide Injection (IGlarLixi). GLP-1 agonists are available in short-acting products, long-acting formulations, and injectable and oral forms (See Table 1, Supplementary Materials).

What can be found is that GLP-1R can promote insulin secretion, suppress appetite, reduce body weight, improve blood lipid profile, and reduce the risk of cardiovascular diseases, which greatly increases the potential of drug development targeting GLP-1R. Targeting GLP-1R is one of the important ways to treat obesity. Not only has a single long-acting GLP-1RA agonist been developed, but a large amount of research and development has also been invested in double and triple GLP-1RA agonists (as shown in Figure 6). For example, Tirzepatide is a dual-target agonist of GIPR and GLP-1R. Insulin Degludec and Liraglutide Injection is a combination of GLP-1RA plus basal insulin in fixed ratios. GLP-1RA and the sodium–glucose cotransporter-2 inhibitor combination. Others include the GLP-1R/GIPR/GCGR triple agonist Retatrutide, etc. These innovative treatment strategies based on GLP-1 receptor agonists (GLP-1RAs) provide a certain guarantee for the healthy life of people with obesity. This article will discuss in detail the application of specific single, double, and triple GLP-1RA agonists in obesity treatment.

### 3.1. Single Agonists

GLP-1 originates primarily in intestinal endocrine L cells and glucagon pre-neurons (named after the transcript) or glucagon neurons (named after the gene) located in the NTS of the posterior brain. Conventional or habitual thinking has assumed that the peripheral (intestinal origin and exogenous origin) and central (brain-derived) GLP-1 systems are interconnected, but current evidence suggests that they are likely to be separate entities (Table 2).

Liraglutide is a GLP-1 receptor agonist that acts on the hypothalamic feeding center to increase satiety signals, reduce appetite, and reduce weight, and can be used for a long time [18,32]. In 2014, Liraglutide 3mg became the first GLP-1-based anti-obesity medications (AOMs) to be introduced to the U.S. market for the treatment of adult obesity and was approved in 2020 for weight management in adolescents with obesity ages 12 and older [33]. The recently FDA-approved 2.4mg dose of Semaglutide reduced average body weight to 68% after 15 weeks of treatment [34], and the drug was well tolerated but still had typical GLP-1-related adverse effects, including nausea, diarrhea, vomiting, and constipation [35]. Some studies have found that in patients with type 2 diabetes, Semaglutide and Liraglutide have a kidney protective effect, which is more obvious in patients with existing chronic kidney disease [36]. It also reduces the risk of cardiovascular disease [37] and reduces visceral fat content [38]. Visceral fat reduction may be a mechanism for the beneficial effects of Liraglutide on cardiovascular outcomes in people with obesity.

Beinaglutide can reduce liver weight and liver steatosis and improve insulin sensitivity [39]. Beinaglutide may be another effective treatment for obesity and NASH. Another GLP-1RA, efpeglenatide, was also found to significantly lower blood sugar and reduce weight, with similar safety and tolerability to other GLP-1RA [40]. Ecnoglutide (XW003), also a GLP-1 peptide analogue, has been found to significantly lower blood glucose, promote insulin induction, and result in more significant weight loss than Semaglutide, with tolerance [41], promoting the continued development of drugs to treat obesity.

GLP-1R peptide agonists have revolutionized obesity and diabetes treatment, but their use is limited due to their need for injections. Therefore, the development of oral bioavailable small molecule GLP-1R agonists is very promising. A daily oral formulation of Semaglutide has recently been approved, and it has demonstrated clinical effectiveness close to that of a once-weekly subcutaneous formulation. Danuglipron, another orally administered small molecule GLP-1R agonist, has also been found to increase insulin levels in primates [42]; it is also effective in weight loss in adults with type 2 diabetes [43]. Orforglipron may provide a safe and effective once-daily oral treatment alternative to injectable GLP-1RAs or peptide oral formulations that are effective in improving blood sugar and significantly reducing body weight [44,45]. In addition, a new GLP-1/Fc fusion protein (TG103) has been developed specifically for the treatment of type 2 diabetes and obesity. It is effective in improving blood sugar and reducing body weight and is well tolerated [46], providing a potential well-tolerated and safe option for the treatment of hypoglycemia.

### 3.2. Double Agonists

The study found that in addition to a single long-acting GLP-1RA agonist, the study found that double and triple gastrointestinal hormone receptor agonists have synergistic benefits in weight loss and improving blood sugar, and single receptor agonists complement each other, exerting a therapeutic effect of 1 + 1 greater than 2. The table below shows the most representative double agonists and triple agonists used to treat obesity in animals and humans (Table 3).

*GLP-1/GIP double-receptor agonist.* In fact, GIP itself is less attractive as a therapeutic target for the treatment of obesity because GIP mainly promotes the storage of TAG after eating through the direct activation of GIPR on adipocytes, indirectly through the lipogenesis of insulin, or through the combination of the two. GIP also improves energy storage by promoting WAT expansion. It has significant pro-insulin activity, but the hypothesis remains that GIP blocking reduces body weight. However, a new study found that the combination of GIP and GLP-1 had a stronger effect on appetite suppression and weight loss than GLP-1Ras alone [50]. GIP may drive weight loss by directly targeting its receptors in the central nervous system to inhibit caloric intake, by enhancing the anorexic effects of GLP-1, or by amplifying the efficacy of GLP-1RA by reducing drug-induced nausea, or a combination of both. In the combination formulation of GLP1R/GIPR, the ability of GIP to improve lipid and glucose metabolism was significantly enhanced, and the two worked together to improve metabolism.

RG-7697, a GIP/GLP-1 double-receptor agonist, has been found to significantly improve blood sugar, reduce body weight, and lower total cholesterol compared to placebo [47,51]. But the most promising GLP-1/GIP double agonist drug is Tirzepatide, which activates the GIP receptor more efficiently than the GLP-1 receptor, an analogue that balances the activity of GLP-1 and GIP receptor agonists, and Tirzepatide has shown powerful advantages in blood sugar control and weight. There is no increased risk of hypoglycemia, and the ability to lower HbA1c is superior to Semaglutide [52,53]. Lilly is currently developing Tirzepatide for the treatment of obesity, T2DM cardiovascular disease, heart failure, NASH, and obstructive sleep apnea [54]. Several other GLP-1R/GIPR double agonists are currently under active development, including CT-388 and CT-868 [2,55]. A drug consisting of a GLP-1R agonist and a GIPR antagonist (AMG133) is also currently in Phase 1 clinical trials [56], and determining whether this mechanism works in humans may take time to study.

*GLP-1/GCG double-receptor agonist*; *OXM analogues (natural GLP-1/GCG receptor agonists)*; *GLP-1/amylin*. Glucagon’s ability to increase energy expenditure and fat oxidation is beneficial in the treatment of fatty diseases and is an attractive co-partner for GLP-1. GLP-1 and glucagon work together to reduce food intake and increase heat production and lipolysis. The GLP-1 and GCG double-receptor agonist, Mazdutide, was found to be effective in reducing body weight and improving blood glucose [57]. Further studies found that Mazdutide in either 9 mg or 10 mg doses was well tolerated and safe [58,59]. Cotadutide is a balanced GLP-1R/GCGR bi-agonist that alleviates NASH and liver fibrosis by regulating mitochondrial function and lipogenesis [60]. Improve blood sugar and weight loss in overweight or patients with obesity by enhancing postprandial insulin secretion and delaying gastric emptying. It was well tolerated [61,62]. Another GLP-1R/GCGR double agonist, SAR425899, has been shown in trials to improve postprandial blood glucose by enhancing beta cell function and slowing glucose absorption [63,64], improving insulin sensitivity and reducing body weight. It also leads to increased lipid oxidation [65], which is beneficial for weight loss and weight maintenance. JNJ-64565111 is also a GLP-1R/GCGR bi-agonist, capable of weight loss in a dose-dependent manner but with a higher incidence of adverse events during treatment [66]. BI456906 is a novel GLP-1R/GCGR double agonist with powerful anti-obesity efficacy [67,68], which is mainly achieved by increasing energy expenditure and reducing food intake [69]. Efinopegdutide has also been found to significantly reduce body weight and liver inflammation in patients with NASH [70]. Efinopegdutide may decrease liver fat content (LFC) indirectly through weight loss or may decrease LFC by acting directly on the liver to stimulate fatty acid oxidation and reduce fat production. Several other GLP-1R/GCGR double agonists are also being actively developed. They contain Pmvidutide [71], NNC9204-1177, OPK88003, MK8521 [55], and MOD-6031 [72].

Oxyntomodulin (OXM) represents a natural double agonist of GLP-1 and glucagon receptors. Its main function is to reduce gastric acid and pancreatic exocrine, suppress appetite and increase energy expenditure, promote lipolysis, and enhance insulin secretion. In addition, people with obesity are more sensitive to OXM and also show good sensitivity and tolerance during long-term use. OXM can cross the blood–brain barrier to reach the appetite center of the hypothalamus and reduce appetite [73]. Some studies have found that OXM can play important physiological roles such as regulating appetite, increasing energy consumption, promoting insulin release, and β cell protection by simultaneously activating GLP-1 receptor and GCGR [74], showing good effects of weight intervention and improved glucose tolerance. An in vitro study found that OXM has a much lower affinity for GLP-1 receptors than GLP-1, while OXM at the same concentration exerts the same appetite-inhibiting effect as GLP-1 [75]. Compared with pure GLP-1R agonists, OXM has shown better efficacy in weight loss and lipid reduction. Weight loss at this time may be mediated by glucagon receptor activation [76], which is closely related to glucagon’s negative energy balance effect of increased thermogenesis, promotion of lipolysis, fatty acid oxidation, and ketone body formation. Some studies suggest that OXM can also exert its food inhibition effect by inhibiting the secretion of ghrelin (the only known gastrointestinal appetite-stimulating hormone), and this process also involves the participation of the GLP-1 receptor [77]. OXM can also increase energy expenditure by upregulating the secretion of insulin, stimulating the synthetic secretion of thyroid hormone, and promoting the breakdown of fat [78]. In obese/overweight patients, the plasma leptin level in the OXM treatment group was significantly lower than that in the control group; the adiponectin level was significantly higher, and the adipose tissue volume was decreased [79]. Therefore, the weight loss after OXM treatment may be due to the loss of fat mass.

In recent years, efforts have been made to develop analogues with similar physiological functions to OXM but more stable pharmacokinetics [78]. One of the OXM analogues (OXM6421) has better GLP-1 receptor affinity and higher anti-degradation enzyme activity than natural OXM. When applied to normal-weight rodents, a single injection can strongly inhibit feeding and increase energy expenditure, and its effect lasts up to 24 h [80]. In mice with obesity, OXM6421 was injected subcutaneously once a day for 21 days, resulting in significant weight loss, along with improved glucose homeostasis and increased circulating adiponectin levels. Another novel chemically modified oxytocin analogue, Oxm [mPEG-PAL], has been shown to improve glucose homeostasis, promote insulin secretion, suppress appetite, and promote fat metabolism [81].

Amylin is an acidic peptide hormone composed of 37 amino acids that are stored and secreted together with insulin, mainly from pancreatic cells [82]. The anaerobic capacity of amylin contributed to the development of Pramlintide, which has been approved by the FDA for use in patients with diabetes alone or in combination with oral medications (insulin, metformin). The combination of Amylin and GLP-1 is expected to yield greater efficacy [83], and the study found that in obese diabetic mice, successive infusions of AC164204 and AC164209 reduced blood sugar and HbA1c more significantly than medication alone [84]. The combination of drugs can be considered when the development of agonists is not good. For example, the long-acting amylin analogue, cagrilintide, has successfully completed the Phase 1b trial [85], and its combination with the GLP-1R agonist Semaglutide, which has the strongest weight loss effect [86], can play a greater role in weight loss with good tolerance and acceptable safety.

*GLP-1/secretin*. Secretin regulates gastric pH and pancreatic bicarbonate secretion, and its effect on brown adipose tissue mediates thermogenesis and stimulates the fat fraction of cultured adipocytes [87]. Acute injection of secretin can activate glucose uptake in human brown adipose tissue. It also enhances arginine by stimulating insulin secretion and reducing blood sugar. Can Secretin/GLP-1 double agonists provide greater weight loss benefits? A study that developed a novel secretin/glucagon-like peptide-1 co-agonist (GUB06-046) found that it can significantly reduce food intake and improve glucose tolerance in mice with diabetes. Long-term administration of GUB06-046 to diabetic db/db mice improved glycemic control, retained β cell quality, and had no effect on the quality of the exocrine pancreas or pancreatic duct epithelium [88].

*GLP-1 and CCK double-receptor agonists*. The combined activation of GLP-1 and CCK receptors may synergically enhance the appetite inhibition and glucose homeostasis effects of a single peptide. At present, studies have characterized an acylated GLP-1/CCK double-acting hybrid peptide [Lys12Pal] Ex-4/CCK (exendin-4). Studies have found that it has a significant insulin-stimulating effect, can regulate glucose homeostasis, and reduce body weight [89]. Another study reported the role of a novel fusion peptide (C2816) consisting of the stable GLP-1R agonist AC3174 and the CCKR selective agonist AC170222 [90]. C2816 retains full activation on GLP-1R and CCKR with lower potency than a single molecule. However, the fusion peptide (pGlu-Gln)-CCK-8/exendin-4 previously reported did not show high activity on any receptor. Although the activity of (pGlu-Gln)-CCK-8/exendin-4 was lower, it could effectively reduce triglyceride and cholesterol levels and improve beta cell area and insulin-stimulating ability, potentially treating metabolic diseases. Compared with single-administration, C2816 showed a better reduction in body weight [91].

*GLP-1/PYY double-receptor agonist*. GLP-1 and PYY, both secreted from L cells in response to food stimulation, have a stronger inhibitory effect on food intake when combined compared to their individual role as a single agonist, with PYY and GLP-1 playing complementary roles [92]. Nausea and vomiting are the main side effects of obesity drugs, and the treatment of obesity based on PYY has found that PYY receptor activation is also associated with nausea and vomiting. When combined with drugs, it may also be necessary to consider that the combination of two drugs can reduce side effects. Studies using GIPR agonists in combination with PYY have found that the combination of GIPR agonists can inhibit PYY-induced nausea [93]. It may be that central and peripheral administration of GIPR agonists reduces conditioned taste avoidance (CTA) without affecting PYY analogues mediated appetite loss. GIPR agonists reduce PYY-mediated neuronal activity in the parabrachial nucleus (PBN), providing a potential mechanistic explanation for how GIPR agonist therapy reduces PYY-induced nausea behavior. Therefore, a new mechanism may have been discovered for a GIP-based therapy to improve the tolerance of weight loss agents.

### 3.3. Triple Agonist

*GLP-1R/GIPR/GCGR triple agonist*. Retatrutide is a novel triple-agonist peptide that acts on GCGR, glucose-dependent insulin-stimulating GIPR, and GLP-1R. Retatrutide showed balanced GCGR and GLP-1R activity but more GIPR activity. Administration of Retatrutide significantly reduced body weight and improved blood glucose control compared with other incretin receptor-targeting molecules [94]. Weight loss was enhanced primarily through increased GCGR-mediated energy expenditure and decreased calorie intake driven by GIPR and GLP-1R [95,96]. In addition, Bossart et al. developed SAR441255, a synthetic peptide agonist for GLP-1, GCG, and GIP receptors based on the exendin-4 sequence [97]. It has high performance and can balance the activation of three receptors. Metabolic outcomes were superior to those in the dual GLP-1/GCG agonist group and improved glycemic control, with an acceptable safety profile in terms of gastrointestinal tolerability and cardiovascular hemodynamics [98]. Other drug candidates include a series of fatty acylated single-molecule GLP1R/GIPR/GCGR tri-agonists. One drug candidate (HM15211) is currently an early clinical trial for the treatment of non-alcoholic steatohepatitis [49], with the ultimate goal of regression of steatohepatitis, improvement in metabolic disease, and treatment of obesity. There are many drug candidates with clinical and preclinical research statuses. The GLP-1/GIP/Glucagon ternary therapy aims to enhance weight loss by adding the benefits of glucagon action to the double effect of GLP-1/GIP. Integrating GIP activity into GLP-1 and GCG receptor agonists may enhance the effect of improved weight loss and glycemic control while buffering the risk of diabetes associated with chronic GCG receptor excitation. It is the future research direction and goal to combine the three with each other and synergistically play a stronger curative effect.

*GLP-1/OXM/PYY:* In addition to GLP-1/GIP/glucagon ternary therapy, another GLP-1/OXM/PYY triple therapy was found. OXM, along with GLP-1 and PYY, are intestinal anorexia hormones secreted from intestinal endocrine L cells. Studies found that postprandial release of GLP-1, OXM, and PYY increased after bariatric surgery, especially after Roux-en-Y gastric bypass (RYGB). Even days after surgery, improved blood sugar control alleviates diabetes remission, as well as weight loss and other beneficial metabolic effects such as enhanced insulin sensitivity and improved lipid profile. Food intake can be reduced by acute continuous subcutaneous infusion of GLP-1, OXM, and PYY after surgery. It achieved superior glucose tolerance and reduced glucose variability [99]. GOP (GLP-1, OXM, and PYY) may be a viable alternative for the treatment of diabetes, which is very beneficial for weight loss [100]. The triple activation of GLP-1, glucagon, and PYY receptors using GLP-1/OXM/PYY combination may be superior to RYGB in metabolism, exploring a new way for future research on obesity drugs.

*GLP-1/GCG/CCK2:* GLP-1 and GCG receptor double agonists have bright prospects and excellent effects in the treatment of obesity and diabetes. GLP-1 and cholecystokinin 2 (CCK2) double agonists can also restore pancreatic function and improve blood sugar. If the two are combined into a triple agonist with both GLP-1/GCG/CCK2 acting, will it produce a more significant anti-obesity and anti-diabetes effect? A novel peptide, Xenopus (x), has been invented, which is an effective and selective triple agonist of GLP-1, GCG, and CCK2 [101]. It was significantly superior to ZP3022 [102] (GLP-1/CCK2 double agonist) and Liraglutide in metabolic effects, weight loss, and liver fat content reduction. It also increases insulin levels and shows more significant and lasting improvements in glucose tolerance and glucose control. Therefore, the GLP-1/GCG/CCK2 triple agonist also has significant potential for the treatment of obesity and diabetes.

## 4. Other Promising Treatments for Obesity

The study of drugs that target key molecules/pathways mediating abnormal states is critical for the treatment of obesity as well as related complications. There are also some drugs being studied to treat obesity by regulating the targets of various systems and tissues, mainly including the central nervous system, peripheral tissues (adipose tissue), peripheral target organs (kidneys, skeletal muscle, liver), and peripheral signaling molecules (gastrointestinal hormones) (Table 4).

In addition, there are many other non-pharmacological strategies to treat obesity. For example, obesity can be treated through gene therapy [29]. Obesity can also be treated by regulating the content of intestinal microbes and improving the distribution of intestinal flora in patients. In recent years, a large number of reports have proved that gut flora is closely related to obesity and plays an important role in regulating host metabolism. Regulation of gut microbiota can increase EEC activity, enhance post-meal satiety secretion, improve glucose and fat metabolism, and increase leptin sensitivity [103,104]. However, the mechanism by which gut microbiota is involved in energy homeostasis remains unclear. However, microbial metabolites can induce epigenetic modifications with potential implications for health status and susceptibility to obesity [11].

Fecal bacteria transplantation (FMT) is a method of transplanting functional bacteria from the stool of a healthy person into the gastrointestinal tract of a patient to reshape the intestinal microbial environment. In recent years, researchers have found that fecal bacteria transplantation can not only reshape the intestinal microecology of obese patients and alleviate weight gain but also be widely used in the validation of natural products targeting intestinal microbiota to treat obesity [105,106]. FMT can alter intestinal flora structure and improve intestinal barrier integrity, thereby improving endotoxemia and insulin resistance [107]. A single dose of oral FMT combined with a daily low-fermentation fiber supplement improved insulin sensitivity in patients with severe obesity and metabolic syndrome and was well tolerated [108]. A large number of studies have found that the effective components and compounds of traditional Chinese medicine can multi-target and comprehensively control intestinal flora, restore bacterial homeostasis, then regulate energy metabolism, inhibit fat accumulation, affect appetite, reduce intestinal mucosal inflammation, and promote weight loss [109,110]. Other studies have found that compound probiotics can alleviate type 2 diabetes in mice by regulating the intestinal microbial community and inducing GLP-1 secretion [111]. All these provide potential strategies for obesity prevention and treatment.

**Table 4:** Drugs to treat obesity.

Agent	Development Stage	Indication	ClinicalTrials.gov ID
**Acts on the central nervous system**			
**Central Nervous System-Secreted Neuropeptides and Antagonists**			
Tesofensine (NS-2330) (serotonin–norepinephrine–dopamine reuptake inhibitor, SNDRI)	Phase 3	Alzheimer’s Disease (AD); Parkinson’s Disease (PD); Obesity; Obesity due to hypothalamic injury	NCT00394667
Oxytocin	Phase 1	Obesity; Adult hypothalamic obesity; Prader–Willi syndrome	NCT02849743
Neuropeptide Y receptors antagonist: Velneperit (S-2367)	Phase 2	Obesity	See Related links [112]
Methylphenidate (Dopamine Reuptake Inhibitor)	Preclinical	Hypothalamic obesity	See Related links [113]
GDF-15	Phase 2	Obesity; Diabetes; Cardiovascular diseases; Systemic lupus erythematosus	NCT00609622
**Endocannabinoid System Agents (Cannabinoid-1 Receptor Antagonists)**			
Rimonabant	Preclinical	Obesity; Cardiovascular risk factors	See Related links [114]
AM4113	Preclinical	Obesity	Not Recorded
JD5037	Preclinical	Obesity	Not Recorded
**Acts on peripheral tissue (adipose tissue)**			
Mirabegron	Phase 3	Overactive bladder; Metabolic disease	NCT02045862 NCT03049462 NCT02919176
**Acts on peripheral target organs (kidney, skeletal muscle, liver)**			
**Sodium–glucose cotransporter2 (SGLT2) inhibitor**			
Empagliflozin (Jardiance)	FDA-approved (2014)	Obesity; T2DM; Heart failure; Chronic Kidney Disease (CKD)	NCT01131676 NCT03485222
Dapagliflozin	Phase 3	T2DM; Heart failure; Chronic Kidney Disease (CKD)	NCT03619213 NCT03036150 NCT03036150
Bexagliflozin	FDA-approved (2023)	T2DM	See Related links [115]
**Dual sodium–glucose cotransporter 1/2 inhibitor (SGLT1/2i)**			
Sotagliflozin	Phase 3	Diabetes; Recent worsening heart failure; Chronic Kidney Disease (CKD)	NCT03521934 NCT03315143
Licogliflozin	Phase 2a	Obesity; NASH	NCT03320941 NCT03205150
**Others**			
Bimagrumab(human monoclonal antibody binding)	Phase 2	Obesity; T2DM; Sarcopenia	NCT03005288 NCT02333331
CRMP (controlled-release mitochondrial protonophore)	Preclinical	Metabolic syndrome; T2DM; NASH; NAFLD	Not Recorded
BAM15	Preclinical	Obesity	Not Recorded
**Act on peripheral signaling molecules (gastrointestinal hormones)**			
**Ghrelin signaling**			
GLWL-01	Phase 2	PWS	NCT03274856
AZP-531	Discontinued	PWS; Obesity; T2DM	See Related links [116]
Spiegelmers NOX-B11	Preclinical	Obesity	Not Recorded
**Leptin sensitizers**			
Celastrol	Preclinical	Obesity; T2DM	See Related links [117]
Setmelanotide (MC4R)	Phase 3	Obesity; T2DM	NCT02896192 NCT03287960
Metreleptin	Phase 3	Lipodystrophy; T1MD; NSAH; Rabson–Mendenhall syndrome (RMS)	NCT01778556 NCT00596934 NCT01679197 NCT00085982 NCT00001987
**GLP2R agonists**			
Teduglutide	Preclinical	Obesity; T2DM	Not Recorded
**PYY analogues**			
NNC0165-1273	Preclinical	Obesity; T2DM	Not Recorded
**OXM**			
OXM 6421	Preclinical	Obesity; T2DM	Not Recorded
**FGF21/FGFR1c/β-Klotho signaling**			
LLF580	Phase 1	Obesity; NASH	NCT03466203
BFKB8488A	Phase 1	Obesity; T2DM; NAFLD	NCT03060538
Pegbelfermin	Phase 2	Obesity; T2DM; NASH	NCT02413372
LY2405319	Phase 1	Obesity; T2DM	See Related links [118]
**Other appetite suppressants**			
Withaferin A	Phase 1	Obesity; T2DM	Not Recorded

## 5. Discussion

With the continuous improvement in people’s living standards and the increasing number of obese people, AOM drugs urgently need to develop rapidly. The identification of GLP-1 drug targets associated with appetite homeostasis provides unprecedented benefits for weight management in obese people, with weight loss of 20% or more seemingly possible. Based on its side effects, the improvement in gastrointestinal reaction and fertility can be studied in the future. GLP-1 targeted drugs can reduce weight by suppressing appetite, increasing energy consumption, and controlling blood sugar. However, at present, there is no research to prove the damage of appetite suppression to brain nerves, and it is not clear whether there are other side effects after long-term use of such drugs. Therefore, this article suggests that double agonists or triple agonists can be developed in combination. The synergistic effect of the two effects plays a stronger role in the same or different targets; when acting on different targets, one target plays a major role but produces certain side effects, and the other target alleviates side effects while playing a role in weight loss, which can reduce the overall side effects. In the research and development of new drug targets for triple agonists, new targets can be added on the basis of double agonists. For example, the inclusion of GIPR targets in GLP-1R/GCGR agonists may buffer the risk of diabetes from chronic GCG receptor excitation.

Research into the development of next-generation drugs is largely hampered by current clinical manifestations and the ability to successfully translate in vitro and animal pharmacology into human trials. In contrast to the registered AOMs, solving the problems described now will be a breakthrough. At present, the number of people with obesity has increased dramatically, and what needs to be achieved most is to speed up safe and normal weight loss. Multi-genomics may be needed to explore some new targets.

The rapid development of science and technology may be more effective in describing the mechanism of preclinical action than in finding clinically successful candidate drugs. What is needed is to optimize the methods of weight loss treatment and speed up drug research and development. Obesity can be prevented in advance if the mechanism of obesity or the mechanism of successful weight loss can be predicted in advance. It may be more beneficial to combine preclinical molecular mechanism research with clinical application exploratory research. Establishing the relationship between obesity and metabolic multi-factors may have profound implications for the future healthcare of obesity.

FMT is a treatment for obesity and metabolic diseases based on intestinal microflora, but whether the native intestinal flora is resistant to the invasion of new species after inhibition is unknown, whether the donor’s flora plays a role or the donor’s flora affects the recipient’s flora plays a role, whether the donor’s flora can be planted in the recipient, and whether the efficacy can be achieved even if it is successfully planted. These are the challenges to be faced.

## 6. Conclusions

Taking GLP-1 as the target, this article expounds on the weight-loss drugs approved and designed based on the GLP-1 target. The article summarizes the characteristics of dual and triple GLP-1 targeted drugs in the treatment of obesity—which yield more benefits and less toxic side effects—and provides a reference for making full use of multi-target methods to treat other diseases in the future. It is innovatively pointed out that intestinal flora and microorganisms have many benefits in the treatment of obesity, and fecal transplantation is a potential treatment method for obesity with little harm to the body and has strong practical value. It is a healthy and promising strategy to treat obesity through multiple GLP-1 targets and changing intestinal microbial structure in the future. Only by improving the limitations of various treatment methods and conducting joint research on obesity mechanisms to explore new treatment methods can more safe and long-term effective treatment strategies be put into practice in the future.

## Figures and Tables

**Table 1:** Approved GLP-1R agonists worldwide.

Agent	Drug Name	Approved Company	Approval Time	Half-Life Period	Administration	Indication	Adverse Reaction
Exenatide	Byetta	Amylin; AstraZeneca	2005.04 (Food and Drug Administration, FDA)	1~2 h	s.c. twice daily	Diabetes mellitus type 2 (T2DM)	Nausea, vomiting, hypoglycemia
Exenatide LAR	Bydureon	Amylin; AstraZeneca	2012.01 (FDA)	5~6 d	s.c. once weekly	T2DM; Parkinson’s disease	Nausea, vomiting, strong immunogenic reactions at the injection site (due to rejection of the polylactic-glycolic acid copolymer)
Liraglutide	Victoza	Novo Nordisk	2010.01 (FDA)	10~14 h	s.c. once daily	Obesity; T2DM; NASH	Nausea, vomiting, excessive weight loss in T2DM patients, hypoglycemic events in combination with conventional hypoglycemic agents
Semaglutide	Ozempic	Novo Nordisk	2017.12 (FDA)	165 h	s.c. once weekly	Obesity	Potential reproductive toxicity
Semaglutide	Rybelsus	Novo Nordisk	2020.01 (FDA)	24h	po. once daily	Obesity	Potential reproductive toxicity
Tirzepatide	Mounjaro	Eli Lilly	2022.05 (FDA)	5~6 d	s.c. once weekly	T2DM	Nausea, diarrhea, decreased appetite, vomiting, constipation, indigestion, stomach pain
Albiglutide	Tanzeum	GSK	2014.04 (FDA)	5~6 d	s.c. once weekly	T2DM	Stop selling
Dulaglutide	Trulicity	Eli Lilly	2014.09 (FDA)	5 d	s.c. once weekly	T2DM	Nausea, vomiting, diarrhea
Lixisenatide	Adlyxin	Sanofi	2016.07 (FDA)	24h	s.c. once daily	T2DM	Fewer side effects
Beinaglutide	HYBR-014	Shanghai Benemae	2023.07 (CFDA)	6h	s.c. thrice daily	Obesity; T2DM; NASH	Widespread antibody
Polyethylene Glycol Loxenatide Injection	PEX-168	Jiangsu Hansoh Pharmaceutical Group	2019.05 (CFDA)	5~6 d	s.c. once weekly	T2DM	The widespread existence of anti-PEG antibodies limits the applicable population
Insulin Degludec and Liraglutide Injection	IDegLira (Xultophy)	Novo Nordisk	2022.03 (FDA)	24h	s.c. once daily	T2DM	Nausea, vomiting, hypoglycemia, injection site reactions
Insulin Glargine and Lixisenatide Injection	IGlarLixi	Sanofi	2023.07 (FDA)	24h	s.c. once daily	T2DM	Nausea, vomiting, hypoglycemia, injection site reactions

**Table 2:** Single target drugs in the research stage.

Agent	Company	Development Stage	Indication	ClinicalTrials.gov ID
Efpeglenatide (LAPSExd4 Analogue)	Hanmi	Phase 3	Humans with obesity and/or T2DM	NCT03353350 NCT03496298 NCT03353350
TG103	CSPC	Phase 2/1	Obesity	NCT03990090
Ecnoglutide (XW003)	Sciwind Biosciences	Phase 2/1	Obesity; T2DM	NCT04389775
Danuglipron (PF-06882961)	Pfizer	Phase 3/2	Obesity; T2DM	NCT03538743
Orforglipron (LY3502970)	Eli Lilly	Phase 2	Obesity; T2DM	NCT05048719
PB-119	PegBio Co.	Phase 2	T2DM	NCT03520972 NCT02084251

**Table 3:** Double and triple target drugs in the research stage.

Agent	Company	Development Stage	Indication	ClinicalTrials.gov ID
**Double agonists**				
**GLP1R/GIPR dual agonists**				
RG-7697 (MAR-709, NNC0090-2746)	F. Hoffmann-La Roche Ag; vo Nordisk	Phase 2	Obesity; T2DM	See Related links [47]
CT-868	Carmot Therapeutics	Phase 2	Obesity; T2DM	Not Recorded
CT-388	Carmot Therapeutics	Phase 1	Obesity; T2DM	Not Recorded
**GLP-1R agonist and GIPR antagonist**				
AMG133	Amgen	Phase 1	Obesity	Not Recorded
**GLP-1R/GCGR dual agonist**				
Mazdutide (IBI362/LY3305677)	Eli Lilly	Phase 1	Obesity; T2DM	NCT04440345
Cotadutide/MEDI0382	AstraZeneca	Phase 2	T2DM	NCT02548585 NCT04208620 NCT03550378
SAR425899	Sanofi	Phase 1	T2DM	NCT02973321 NCT02411825
JNJ-64565111	Johnson	Phase 2	Obesity; T2DM	NCT03586830 NCT03486392
BI 456906	Boehringer Ingelheim	Phase 2 Phase 1	Obesity	NCT03175211 NCT03591718
Efinopegdutide	Hanmi Pharmaceutical	Phase 2	NASH	NCT04944992
Pemvidutide/ALT-801	Altimmune	Phase 1	Obesity; NASH	NCT00496860
JNJ-54728518	Janssen Research & Development Llc	Phase 1	Obesity; T2DM	Not Recorded
NN9277/NNC9204-1177	Novo Nordisk	Phase 1	Obesity	NCT02941042
MOD-6031	OPKO Health	Phase 1	Obesity; T2DM	Not Recorded
OPK-88003	OPKO Health	Phase 2	Obesity; T2DM	Not Recorded
MK8521	Merck Sharp & Dohme Corp	Preclinical	Obesity; T2DM	Not Recorded
**GLP-1/amylin**				
GLP1RA/davalintide hybrid peptides (AC164204, AC164209)		Preclinical	DIO rats and ob/ob mice	Not Recorded
Cagrilintide (NNC0174-0833)	Novo Nordisk	Phase 1	Humans with obesity or overweight	NCT03600480
**GLP-1/secretin**				
GUB06-046	Gubra ApS	Preclinical	Diabetic mice	Not Recorded
**GLP-1/CCK**				
CCK-8/exendin-4 hybrid peptide		Preclinical	DIO mice	Not Recorded
Fusion peptide C2816		Preclinical	DIO mice	Not Recorded
**GLP-1/PYY**				
PYY3-36 + GLP-1		Preclinical	Obesity	See Related links [48]
**Triple agonists**				
**GLP-1R/GIPR/GCGR triple agonist**				
HM15211	Hanmi Pharmaceutical	Phase 2	NASH	See Related links [49]
Retatrutide (LY3437943)	Eli Lilly	Phase 2	Obesity; T2DM	NCT04881760 NCT04867785 NCT04143802 NCT04881760
SAR441255	Sanofi	Phase 1	Obesity	NCT04521738
NN9423/NNC9204-1706	Novo Nordisk	Phase 1	Obesity	NCT03095807

## Supplementary Materials

The following supporting information can be downloaded at: https://www.mdpi.com/article/10.3390/ijms26041651/s1. References [5,34,39,119,120,121,122,123,124,125,126,127,128,129,130,131,132] are cited in the Supplementary Materials.

## Abbreviations

MASH/NASH	non-alcoholic steatohepatitis
MAFLD/NAFLD	non-alcoholic fatty liver disease
T2DM	type 2 diabetes mellitus
PKA	protein kinase A
PI3K	phosphatidylinositol-3-kinase
AKT/PKB	protein Kinase B
AMPK	adenosine 5‘-monophosphate (AMP)-activated protein kinase
MAPK	mitogen-activated protein kinase
PPARγ	peroxisome proliferator-activated receptor γ
FABP4	fatty acid-binding protein 4
BMP4	bone morphogenetic protein 4
UCP1	uncoupling protein 1
ARC	activity-regulated cytoskeleton-associated protein
AgRP	agouti-related protein
NPY	neuropeptide Y
GIP	gastric inhibitory polypeptide
GIPR	gastric inhibitory polypeptide receptor
GCG	glucagon
GCGR	glucagon receptor
NTS	nucleus tractus solitarius
AOM	azoxymethane
FDA	food and drug administration
TAG	triacylglycerol
WAT	white adipose tissue
HbA1c	glycosylated hemoglobin, type A1C
CCK	cholecystokinin
PYY	peptide YY
mTOR	mammalian target of rapamycin
mTORC1	mechanistic target of rapamycin complex 1
mTORC2	mechanistic target of rapamycin complex 2
GLUT4	glucose transporter 4
GSK3	glycogen synthase kinase-3
MAPKKK	mitogen-activated protein kinase kinase kinase
BAT	brown adipose tissue
p38-MAPK	p38 mitogen-activated protein kinase
ERK	extracellular signal-related kinases
ERK5	extracellular signal-related kinases 5
ERK1/2	extracellular signal-related kinases 1/2
TCF	transcription factor
C1EBPα/CCAAT	enhancer-binding proteins
ADD1	adducin 1
APM	acute poliomyelitis
GSK3β	glycogen synthase kinase 3 β
OSBPL2	oxysterol binding protein-like 2
CXXC5	CXXC finger protein 5
NOTUM	notum palmitoleoyl-protein carboxylesterase
