# Supplementary material for: Focus on Glucagon-like Peptide-1 Target: Drugs Approved or Designed to Treat Obesity
Source: Int J Mol Sci. 2025 Feb 14;26(4):1651. doi: 10.3390/ijms26041651 (PMC11855704; doi:10.3390/ijms26041651)
Supplement: Supplementary file 1 [file ijms-26-01651-s001.zip › ijms-3439982-supplementary.pdf]

Available GLP-1 agonists are available in short-acting products, long-acting formulations, injectable and oral forms. (Related to Table.1)

Natural GLP-1RA can be rapidly enzymized by DPP-4 and cannot play a direct role as a drug. In 1992, scientists found Exenatide in Gila monster venom, which has 53% homology with GLP-1(7-37), and 8 amino acids in the middle position are the same as GLP-1, which is the key to the efficacy of GLP-1[1]. It is able to resist DPP4 enzymatic hydrolysis and has a low human kidney clearance, becoming the first GLP-1 drug successfully developed[2]. After the launch of Exentide, the research and development of GLP-1 drugs was mainly directed in two directions: the development of long-acting GLP-1RA and oral GLP-1RA, and various long-acting GLP-1 drugs were born in accordance with the birth.

Liraglutide replaces arginine at position 34 with lysine in GLP-1 molecule, and adds a glutamic acid-mediated 16-carbon palmitic acid side chain to lysine at position 6; Its structure has 97% homology with GLP-1 molecule, and it can strongly resist the degradation of DPP-4 and play a sustained release role[3]. On March 5, 2022, Novo Nordisk launched the world's first basal Insulin GLP-1RA Injection (Insulin Degludec and Liraglutide Injection[4]).

Exenatide LAR(Bydureon) is a sustained release formulation of Byetta, which uses polylactic acid-glycolic acid copolymer (PLGA) to wrap exenatide for sustained release[5]. Bydureon is equivalent to the improvement and innovation of the pharmaceutical preparations already on the market. The new pharmaceutical preparations have changed in the composition of excipients, process, administration frequency, etc., which has a certain impact on the efficacy. The pharmaceutical preparations can be further improved to improve the bioavailability of drugs.

Albiglutide, the second long-acting GLP-1 approved by the FDA in 2014, has 97% homology and is formed by fusing two [Gly8] GLP-1 (7-36) with human albumin to enhance resistance to DPP-4. In clinical trials of Liraglutide (1.8 mg) and Albiglutide (50 mg), Liraglutide was superior for lowering HbA1c, making it easier for patients to lose weight[6]. Albiglutide has fewer hypoglycemic events and moderate weight loss, but Albiglutide based on fusion protein technology has more antibodies due to its functional protein domain[7]. Albiglutides were declared in accordance with BLA, and no excessive studies on metabolism, distribution, excretion, or genotoxicity were carried out. Most importantly, no carcinogenicity tests were conducted; The drug was discontinued in August 2017 due to too few prescriptions.

Dulaglutide (Trulicity) is a long-acting GLP-1RAs, which is mainly covalently linked by GLP-1 analogues and human immunoglobulin IgG 4-heavy chain molecules, which can reduce immunogenicity, enhance water solubility, maintain 90% sequence similarity, and avoid degradation by DPP-4[8, 9]. However, because Dulaglutide has a certain functional protein domain, more antibodies will appear in the body.

Lixisenatide (Adlyxin) is similar in structure to exendin-4, has unique pharmacokinetics, a relatively short half-life, but a strong ability to delay gastric emptying, and has 4 times the affinity for GLP-1 receptors than natural GLP-1[10]. Compared with Exenatide, Lixisenatide was generally well tolerated, with a low incidence of hypoglycemia, low immune response at the injection site, and fewer side effects such as nausea and vomiting[11].

Semaglutide (Ozempic) is the seventh GLP-1RAs approved by the U.S. FDA and has 94% homology with natural GLP-1[12]. On September 20, 2019, the FDA approved the once-daily oral version of Semaglutide (Rybelsus), which uses 2-hydroxybasic formamide as an absorbent to increase the absorption of Semaglutide in the stomach epithelium, improve bioavailability, and get rid of the inconvenience and psychological suffering caused by injection[13]. In Rybelsus' non-clinical studies,

Semaglutide is not the core, but SNAC, a new absorption-enhancing excipient, is the protagonist. Semaglutide was found to be more effective than Liraglutide in dose-dependent reduction of glycosylated hemoglobin (HbA1c) and weight loss[14].

Beinaglutide (HYBR-014) is the world's first GLP-1 drug with a personal amino acid sequence. The drug has a short half-life and needs to be administered with meals to simulate the natural GLP-1 secretion pattern, which is more in line with the physiological rhythm of normal people[15]. However, it is painful for diabetic patients who often need needles, which seriously affects patient compliance. For people of reproductive age, the FDA recommends that Semaglutide not be used during pregnancy due to its potential reproductive toxicity. Beinaglutide has shown the best safety at the level of reproductive toxicity, and it is expected to occupy this segment of the childbearing age population with high safety requirements with its differentiated advantages in safety.

Tirzepatide (Mounjaro) is a dual-target agonist of GIPR and GLP-1R, and its pharmacodynamics mainly targets the combination and activation of the two targets[16]. Polyethylene Glycol Loxenatide Injection (PEX-168) is a new hypoglycemic drug formed by modifying amino acids on the basis of the chemical structure of Exenatide and by the modification of polyethylene glycol[17]. However, due to the existence of anti-PEG antibody and PEG complement activation in vivo, the applicable population is limited.

[1] DONNELLY D. The structure and function of the glucagon-like peptide-1 receptor and its ligands [J]. Br J Pharmacol, 2012, 166(1): 27-41.
[2] XU Q, ZHANG X, LI T, et al. Exenatide regulates Th17/Treg balance via PI3K/Akt/FoxO1 pathway in db/db mice [J]. Mol Med, 2022, 28(1): 144.
[3] LUNDGREN J R, JANUS C, JENSEN S B K, et al. Healthy Weight Loss Maintenance with Exercise, Liraglutide, or Both Combined [J]. N Engl J Med, 2021, 384(18): 1719-30.
[4] HARRIS S, ABRAHAMSON M J, CERIELLO A, et al. Clinical Considerations When Initiating and Titrating Insulin Degludec/Liraglutide (IDegLira) in People with Type 2 Diabetes [J]. Drugs, 2020, 80(2): 147-65.
[5] LI T, CHANDRASHEKAR A, BEIG A, et al. Characterization of attributes and in vitro performance of exenatide-loaded PLGA long-acting release microspheres [J]. Eur J Pharm Biopharm, 2021, 158: 401-9.
[6] NAUCK M A, QUAST D R, WEFERS J, et al. GLP-1 receptor agonists in the treatment of type 2 diabetes - state-of-the-art [J]. Mol Metab, 2021, 46: 101102.
[7] HERNANDEZ A F, GREEN J B, JANMOHAMED S, et al. Albiglutide and cardiovascular outcomes in patients with type 2 diabetes and cardiovascular disease (Harmony Outcomes): a double-blind, randomised placebo-controlled trial [J]. Lancet, 2018, 392(10157): 1519-29.
[8] GERSTEIN H C, COLHOUN H M, DAGENAIS G R, et al. Dulaglutide and cardiovascular outcomes in type 2 diabetes (REWIND): a double-blind, randomised placebo-controlled trial [J]. Lancet, 2019, 394(10193): 121-30.
[9] ARSLANIAN S A, HANNON T, ZEITLER P, et al. Once-Weekly Dulaglutide for the Treatment of Youths with Type 2 Diabetes [J]. N Engl J Med, 2022, 387(5): 433-43.
[10] PFEFFER M A, CLAGGETT B, DIAZ R, et al. Lixisenatide in Patients with Type 2 Diabetes and Acute Coronary Syndrome [J]. N Engl J Med, 2015, 373(23): 2247-57.
[11] KRISTENSEN S L, RøRTH R, JHUND P S, et al. Cardiovascular, mortality, and kidney outcomes with GLP-1 receptor agonists in patients with type 2 diabetes: a systematic review and meta-analysis of cardiovascular outcome trials [J]. Lancet Diabetes Endocrinol, 2019, 7(10): 776-85.
[12] WEGHUBER D, BARRETT T, BARRIENTOS-PéREZ M, et al. Once-Weekly Semaglutide in Adolescents with Obesity [J]. N Engl J Med, 2022, 387(24): 2245-57.
[13] KNOP F K, ARODA V R, DO VALE R D, et al. Oral semaglutide 50 mg taken once per day in adults with overweight or obesity (OASIS 1): a randomised, double-blind, placebo-controlled, phase 3 trial [J]. Lancet, 2023, 402(10403): 705-19.
[14] RUBINO D M, GREENWAY F L, KHALID U, et al. Effect of Weekly Subcutaneous Semaglutide vs Daily Liraglutide on Body Weight in Adults With Overweight or Obesity Without Diabetes: The STEP 8 Randomized Clinical Trial [J]. Jama, 2022, 327(2): 138-50.
[15] FANG X, DU Z, DUAN C, et al. Beinaglutide shows significantly beneficial effects in diabetes/obesity-induced nonalcoholic steatohepatitis in ob/ob mouse model [J]. Life Sci, 2021, 270: 118966.
[16] FRANCE N L, SYED Y Y. Tirzepatide: A Review in Type 2 Diabetes [J]. Drugs, 2024.
[17] WU Y, GUO Z, WANG J, et al. Polyethylene Glycol Loxenatide (PEX-168) Reduces Body Weight and Blood Glucose in Simple Obese Mice [J]. Int J Endocrinol, 2021, 2021: 9951463.
